# A Total Lp-Norm Optimization for Bearing-Only Source Localization in Impulsive Noise with *SαS* Distribution

**DOI:** 10.3390/s21196471

**Published:** 2021-09-28

**Authors:** Ji-An Luo, Chang-Cheng Xue, Ying-Jiao Rong, Shen-Tu Han

**Affiliations:** 1 School of Automation, Hangzhou Dianzi University, Hangzhou 310018, China; luojian@hdu.edu.cn (J.-A.L.); xcc@hdu.edu.cn (C.-C.X.); hanshentu@hdu.edu.cn (S.-T.H.); 2 Science and Technology on Near-Surface Detection Laboratory, Wuxi 214035, China

**Keywords:** bearing-only, source localization, robust estimation, least Lp-norm, total Lp-norm optimization

## Abstract

This paper considers the problem of robust bearing-only source localization in impulsive noise with symmetric α-stable distribution based on the Lp-norm minimization criterion. The existing Iteratively Reweighted Pseudolinear Least-Squares (IRPLS) method can be used to solve the least LP-norm optimization problem. However, the IRPLS algorithm cannot reduce the bias attributed to the correlation between system matrices and noise vectors. To reduce this kind of bias, a Total Lp-norm Optimization (TLPO) method is proposed by minimizing the errors in all elements of system matrix and data vector based on the minimum dispersion criterion. Subsequently, an equivalent form of TLPO is obtained, and two algorithms are developed to solve the TLPO problem by using Iterative Generalized Eigenvalue Decomposition (IGED) and Generalized Lagrange Multiplier (GLM), respectively. Numerical examples demonstrate the performance advantage of the IGED and GLM algorithms over the IRPLS algorithm.

## 1. Introduction

Bearing-Only Source Localization (BOSL) using spatially distributed sensors can be widely applied in network localization [1], vehicle localization [2], gunshot localization [3], animal behavior monitoring [4] and rigid body localization [5], to name but a few. The problem of BOSL is to estimate the source location from a set of noise-corrupted bearing measurements where its main challenge originates in the highly nonlinear nature of the angle observations with the true source position. Under the assumption of Gaussian measurement noise, many estimation approaches have been proposed to handle the nonlinearity, including the grid search method, the pseudolinear estimators [6,7], the iterative maximum likelihood methods [8] and the subspace approaches [9]. However, in the complex field environment, the sensor is vulnerable to external interference, enemy attack or node failure. The bearing measurements may suffer impulse noise [10,11,12,13] and those outlier data can degrade the localization performance dramatically. Therefore, it is necessary to develop new estimators that are robust to impulsive noise.

In fact, non-Gaussian models corresponding to impulsive noise have been extensively studied in the literature [13,14,15]. These studies have demonstrated that the Symmetric α-Stable (SαS) distribution is more suitable to model impulsive noise than the Gaussian distribution. The family of stable distribution is a generalization of the Gaussian distribution under the stable law, including a large range of distributions with mutable values of impulsiveness (α), skewness (β) and dispersion (γ). In particular, two special cases of α-Stable distribution can be obtained by letting impulsiveness parameter α take values of 1 and 2. One is Cauchy distribution (α=1), and the other is Gaussian distribution (α=2).

In the present study, we focus on the problem of robust BOSL with impulsive noise modeled as SαS distribution. In this situation, the methods derived using L2-norm optimization exhibit unreliable estimates because they are not robust to outliers. Therefore, robust statistics need to be used to improve the localization performance. Under the assumption of SαS measurement noise, a Maximum Likelihood Estimator (MLE) was proposed in [16] to produce optimal estimates. Unfortunately, no closed-form expressions exist for the general likelihood function in the cases of 1<α<2 and therefore no explicit solution for MLE is possible. Although the SαS distribution does not have finite variance, it has finite Fractional Lower Order Moments (FLOM) [13], which can be calculated by its dispersion γ and its characteristic exponent α. Moreover, minimizing the FLOM of estimation errors is equivalent to minimizing the dispersion. It is well known as the minimum dispersion criterion, which minimizes the Lp-norm of the estimation residuals. Unlike L2-norm minimization, the least Lp-norm estimator (1<p<2) does not have a closed-form solution and consequently needs to be solved in an iterative manner [17].

The least Lp-norm estimator belongs to the M-estimator. The main idea of M-estimate is to replace the sum of squares of least squares residuals with cost functions more robust to impulsive noise, so as to reduce the sensitivity of estimators with respect to model errors. These functions include Huber [18], Bi-square [19], the negative log-likelihood of the Cauchy distribution [20], Wilcoxon [21], L1-norm [22,23], Lp-norm [24] and L*∞*-norm [25], etc. Ref. [19] presented a distributed robust localization algorithm based on energy information for sensor networks. The algorithm uses Bi-square function as the cost function of M-estimate. A distributed incremental least mean square algorithm based on Wilcoxon norm was proposed in [21] for parameter estimation of sensor networks. Ref. [26] proposed a robust structure total least-squares algorithm for BOSL by using the improved Danish weight function to suppress the impact of outlier data on the localization performance. In addition to the M-estimator, there are other algorithms that can handle outlier data, such as outlier detection [27], clustering [28] and game theoretic techniques [29]. The outlier detection method [27] is to detect suspected outlier data first, and separate it from the original data set. The clustering based techniques [28] can be used to classify normal and abnormal sensors. In game theory [29], the defense strategies could be employed to detect the outlier data and adaptive threshold selection can be also introduced.

Recently, an Iteratively Reweighted Pseudolinear Least-Squares (IRPLS) method was proposed in [14] to reduce biases attributed to the impulsive noise. However, IRPLS still suffers from a major bias problem caused by the correlation between system matrix and the noise vector. This bias can be reduced by exploiting the use of an Instrumental-Variable (IV) matrix [14] that is approximately uncorrelated with the noise vector. Inconsistent with the IV method, we present a robust total least-squares method using Lp-norm minimization that can reduce biases by minimizing the errors in the system matrix and the data vector simultaneously. We first formulate the problem of BOSL subjected to impulsive noise modeled as SαS distribution and review the pseudo-linear measurement model for BOSL. Next, we present the Total LP-norm Optimization (TLPO) method for BOSL to minimize the the errors in the system matrix and the data vector under the minimum dispersion criterion. Two algorithms, named Iterative Generalized Eigenvalue Decomposition (IGED) and Generalized Lagrange Multiplier (GLM), are designed to solve the TLPO problem. The performance advantage of the proposed algorithms is demonstrated by numerical simulations in terms of both bias and Root-Mean-Square-Error (RMSE) performance. The main contributions of the proposed method can be summarized as follows:

- Development of a new bias reduced estimator based on TLPO for BOSL when the measurement noise is modeled as SαS distribution;
- Development of two algorithms for TLPO optimization using the IGED approach and the GLM method, respectively.

The rest of this paper is organized below. Section 2 briefly summarizes the SαS distribution, presents the measurement model for BOSL and discusses the nonlinear least Lp-norm for BOSL. In Section 3, the pseudolinear estimator and the iteratively reweighted pseudolinear least-squares algorithm are reviewed. In Section 4, a new TLPO method is presented, and two iterative algorithms are developed to solve the TLPO problem. Section 5 presents the Cramér-Rao Lower Bound and Section 6 illustrates the bias and RMSE performance of PLE, TLS, LAR and TLAR using various numerical examples. Lastly, conclusions are drawn in Section 7.

## 2. Lp-Norm Optimization for Robust BOSL

### 2.1. Symmetric Alpha-Stable Distribution

The impulsive noise is more likely to exhibit outliers than normal noise. Studies [13] have shown that SαS distribution can model the impulsive noise better than Gaussian distribution due to the reason that the SαS densities have heavier tails than the Gaussian density. The characteristic function of SαS distribution is described as:

(1) $$\chi \left(\tau \right)=\exp\{i\delta \tau -\gamma |\tau {|}^{\alpha }\}$$

where α (0<α≤2) denotes the characteristic exponent, γ indicates the dispersion parameter and δ stands for the location parameter. The SαS distribution is completely determined by these three parameters. The value of α indicates the heaviness of the tails of the density function. A small positive value of α implies high impulsiveness, while a value of α close to 2 shows a type of Gaussian-like shape. The dispersion γ performs like the variance and δ controls the location of the density function.

A SαS distribution is called standard if δ=0, γ=1. Let *x* be a random variable that follows the standard SαS distribution. By taking the inverse Fourier transform of its characteristic function, the density function of *x* is of the form:

(2) $$f_{\alpha }\left(x\right)=\frac{1}{2\pi }\int_{-\infty }^{+\infty }\exp\left(-ix\tau -{\gamma |\tau |}^{\alpha }\right)d\tau$$

It is known that a SαS distribution with characteristic exponent α only has finite moments for orders less than α, which are called the Fractional Lower Order Moments (FLOM). In particular, the FLOM of a standard SαS random variable *x* is given by

(3) $${E\{|x|}^{p}\}=C\left(p,\alpha \right)\gamma^{p/\alpha },\;0<p<\alpha$$

where E{·} denotes expectation operator,

(4) $$C\left(p,\alpha \right)=\frac{2^{p+1}\Gamma \left(\frac{p+1}{2}\right)\Gamma \left(-\frac{p}{\alpha }\right)}{\alpha \sqrt{\pi }\Gamma \left(-\frac{p}{2}\right)}$$

is a constant that depends only on *q* and α and $\Gamma \left(a\right)=\int_{0}^{+\infty }y^{\left(a-1\right)}\exp\left(-y\right)dy$ is the Gamma function. It is worth mentioning that the linear space of a SαS process is a Banach space for α∈[1,2), and it is only a metric space for α∈(0,1) [30]. Therefore, the tools of Hilbert space are not applicable when one solves a linear estimation problem with the SαS process. In the present study, we only focus on the case of α∈(1,2).

### 2.2. Measurement Model

**Figure 1:**
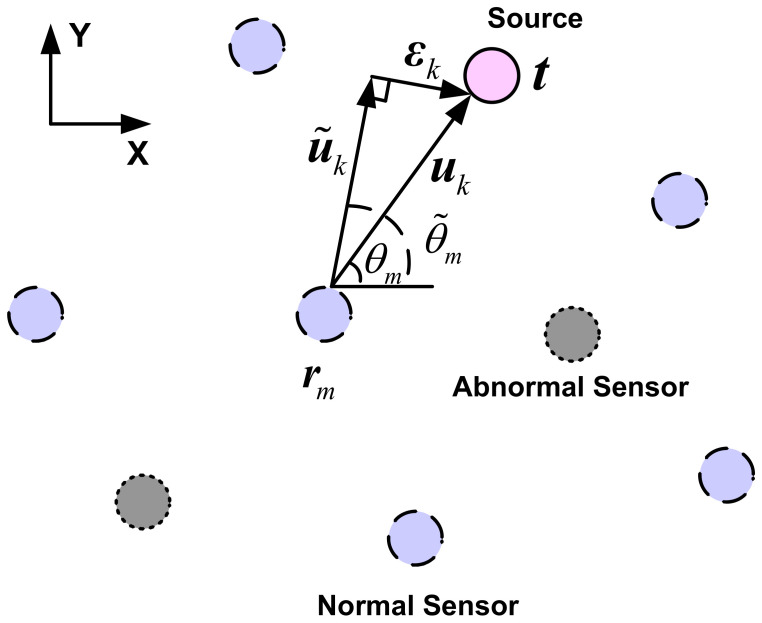
Illustration of the BOSL system.

The problem of robust BOSL is depicted in Figure 1, where $t={\left[t_{x},t_{y}\right]}^{T}$ denotes the unknown target location vector, $r_{m}={\left[r_{x,m},r_{y,m}\right]}^{T}$ represents the *m*th sensor location vector and $\theta_{m}$ is the true bearing at sensor *m*. The nonlinear relationship between $\theta_{m}$, t and $r_{m}$ is given by [31]

(5) $$\theta_{m}=\tan^{-1}\left(t_{y}-r_{y,m},t_{x}-r_{x,m}\right),\;\theta_{m}\in \left[0,2\pi \right)$$

where $\tan^{-1}$ denotes the two-argument inverse tangent function and m=1,2,…,M.

The bearing measurement taken at sensor *m* is given by

(6) $${\tilde{\theta }}_{m}=\theta_{m}+n_{m}$$

where $n_{m}$ follows independent zero mean SαS distribution with $\gamma_{m}$ representing the dispersion parameter and α accounting for the characteristic exponent. The dispersion $\gamma_{m}$ can vary with *m*. If the noise dispersion $\gamma_{m}$ is known *a priori*, then the noise term $n_{m}$ can be normalized. Let $e_{m}=\gamma_{m}^{-1/\alpha }n_{m}$ denote the normalized noise that has unit dispersion. The normalized measurement equation can be written as

(7) $${\tilde{\phi }}_{m}=\phi_{m}+e_{m}$$

where ${\tilde{\phi }}_{m}=\gamma_{m}^{-1/\alpha }{\tilde{\theta }}_{m}$ is the normalized bearing measurement and $\phi_{m}=\gamma_{m}^{-1/\alpha }\theta_{m}$ is the normalized true bearing. Stacking (7) in the vector form yields

(8) $$\tilde{\phi }=\phi +e$$

where $\tilde{\phi }={\left[{\tilde{\phi }}_{1},{\tilde{\phi }}_{2},\ldots ,{\tilde{\phi }}_{M}\right]}^{T}$ denotes the normalized measurement vector, $\phi ={\left[\phi_{1},\phi_{2},\ldots ,\phi_{M}\right]}^{T}$ represents the true bearing vector and $e={\left[e_{1},e_{2},\ldots ,e_{M}\right]}^{T}$ indicates the measurement noise vector.

### 2.3. Minimum Dispersion Criterion

The main difficulty for parameter estimation with the α-stable process is that all non-Gaussian α-stable distributions have infinite variance. As such, the traditional nonlinear least-squares techniques [32], which rely on the second order moments, are not suitable for solving BOSL problems with impulsive noise. Fortunately, we can use the minimum dispersion criterion instead of minimizing the variance. To do this, we first define the norm of the SαS random variable *x* as

(9) $${∥x∥}_{\alpha }=\gamma^{\frac{1}{\alpha }},\;\alpha \in \left[1,2\right)$$

Thus, a suitable measure of the dispersion could be $\gamma ={∥x∥}_{\alpha }^{\alpha }$. Combining (3) and (9), one can easily obtain

(10) $${\left(\frac{E\{|x{|}^{p}\}}{C(p,\alpha )}\right)}^{\frac{1}{p}}\equiv {∥x∥}_{\alpha },\;p\in \left(0,\alpha \right),\;\alpha \in \left[1,2\right)$$

Therefore, we may estimate the source location by solving the following nonlinear Lp-norm optimization problem:

(11) $$\hat{t}=\arg\min_{t}J\left(t\right),\;J\left(t\right)=∥\tilde{\phi }{-\phi ∥}_{p}^{p}=\sum_{m=1}^{M}{\left|{\tilde{\phi }}_{m}-\phi_{m}\left(t\right)\right|}^{p}$$

where p∈(1,α). The definition of the Lp-norm of the vector ζ is

(12) $${∥\zeta ∥}_{p}={\left(\sum_{k}{\left|\zeta_{k}\right|}^{p}\right)}^{1/p}$$

In the present study, we do not consider the case of 0<p≤1, since the corresponding Lp-norm cost function is not differentiable. For non-normalized bearing measurements, the Lp-norm objective function in (11) becomes

(13) $$J\left(t\right)=\sum_{m=1}^{M}{\left|\gamma_{m}^{-1/\alpha }\left({\tilde{\theta }}_{m}-\theta_{m}\left(t\right)\right)\right|}^{p}$$

The optimization problem listed in (11) can be solved numerically for a given 2D space of interest. To be more specific, we can perform a grid search over that 2D space. The global solution of maximum likelihood (ML) estimator is guaranteed for the given set of data as long as the spacing between grids is small enough. However, if the range of the parameter of interest is not confined to a relatively small interval or the dimension of unknown parameter vector is high, the grid search approach is computationally infeasible. Instead, one may resort to iterative optimization methods, such as gradient decent, and Gauss–Newton [33], etc. In particular, the Gauss–Newton algorithm is

(14) $${\hat{t}}^{\left(i+1\right)}={\hat{t}}^{\left(i\right)}-{\left(\nabla J{\left(t\right)}^{T}\nabla J\left(t\right)\right)}^{-1}\nabla J{\left(t\right)}^{T}\left(\tilde{\phi }-\phi \left({\hat{t}}^{\left(i\right)}\right)\right)$$

where ∇J(t) denotes the Jacobian matrix,

(15) $$\nabla J\left(t\right)=p\sum_{m=1}^{M}\frac{\partial \phi_{m}\left(t\right)}{\partial t}{\left|e_{m}\right|}^{p-1}\mathrm{sign}\left(e_{m}\right)$$

If $e_{m}>0$, $\mathrm{sign}(e_{m})=1$ and −1 otherwise. The statistical properties of the nonlinear Lp-norm minimizer have been studied in the literature [24]. The theoretical covariance B is related to the value of *p*, and it is given by [24]:

(16) $$B\approx \frac{C(2p-2,\alpha )}{{\left(p-1\right)}^{2}C^{2}\left(p-2,\alpha \right)}{\left(\nabla \phi^{T}\nabla \phi \right)}^{-1}$$

where ∇ϕ denotes the Jacobian matrix of ϕ with respect to t. By minimizing the scalar term of B, we obtain the optimal choice of *p*

(17) $$p^{o}=\arg\min_{p}\frac{C(2p-2,\alpha )}{{\left(p-1\right)}^{2}C^{2}\left(p-2,\alpha \right)}$$

where $p^{o}$ denotes the optimal value of *p*.

## 3. Pseudolinear Lp-Norm Minimization

### 3.1. Pseudolinear Estimator

The robust BOSL problem is nontrivial because the measurement Equation in (7) is nonlinearly related to the unknown source location. An attractive solution is to set up a pseudolinear equation by lumping the nonlinearities into the noise term. As illustrated in Figure 1, an orthogonal vector sum between the measured angle vector and the true angle vector can be geometrically described by

(18) $$\begin{array}{c} u_{m}={\tilde{u}}_{m}+\varepsilon_{m}=t-r_{m} \end{array}$$

where $u_{m}$ is the true angle vector between $r_{m}$ and t, ${\tilde{u}}_{m}$ is the measured angle vector starting from $r_{m}$ and produces the noisy bearing ${\tilde{\theta }}_{m}$ with respect to the horizontal direction and $\varepsilon_{m}$ is the error vector. Let $\mu_{m}={\left[\cos{\tilde{\theta }}_{m},\sin{\tilde{\theta }}_{m}\right]}^{T}$ and $\nu_{m}={\left[\sin{\tilde{\theta }}_{m},-\cos{\tilde{\theta }}_{m}\right]}^{T}$ denote two orthogonal unit trigonometric vectors. Then, ${\tilde{u}}_{m}$ and $\varepsilon_{m}$ are given by

(19) $$\begin{array}{cc} {\tilde{u}}_{m} & =∥u_{m}∥\cos n_{m}\cdot \mu_{m} \end{array}$$

(20) $$\begin{array}{cc} \varepsilon_{m} & =∥u_{m}∥\sin n_{m}\cdot \nu_{m} \end{array}$$

where ∥·∥ denotes Euclidean norm. Note from (19) that ${\tilde{u}}_{m}^{T}\nu_{m}=0$. Substituting (19) and (20) into (18) and multiplying (18) with $\nu_{m}^{T}$ yields

(21) $$\begin{array}{c} \xi_{m}=\nu_{m}^{T}t-\nu_{m}^{T}r_{m} \end{array}$$

where $\xi_{m}=∥u_{m}∥\sin n_{m}$ is a nonlinear transformed measurement error. Collecting the pseudolinear equation errors as a vector $\xi ={\left[\xi_{1},\xi_{2},\ldots ,\xi_{K}\right]}^{T}$, we obtain

(22) $$\begin{array}{c} \xi =At-b \end{array}$$

where $A={\left[\nu_{1},\nu_{2},\ldots ,\nu_{M}\right]}^{T}$, $b={\left[\nu_{1}^{T}r_{1},\ldots ,\nu_{M}^{T}r_{M}\right]}^{T}$ are the measurement matrix and vector, respectively.

The PLE requires that t be estimated by minimizing ${∥At-b∥}_{2}^{2}$ with respect to t in an L2-norm optimization sense, and the solution is

(23) $$\begin{array}{c} {\hat{t}}_{\mathrm{PLE}}={\left(A^{T}A\right)}^{-1}A^{T}b \end{array}$$

The above PLE solution has a large bias because the abnormal nodes can produce significantly large bearing measurement errors. As a typical robust estimation algorithm, Lp-norm minimization has been widely used for parameter estimation using measurements corrupted by impulse noise. In this paper, we aim to develop robust estimators using Lp-norm optimization to reduce the bias as much as possible.

### 3.2. Iteratively Reweighted Pseudolinear Least-Squares Algorithm

This subsection reviews an Iteratively Reweighted Pseudolinear Least-Squares (IRPLS) algorithm, which has been proposed in [14]. The IRPLS algorithm is derived from the following Lp-norm optimization problem:

(24) $$\min_{t}{∥G\left(At-b\right)∥}_{p}^{p}=\min_{t}\sum_{m=1}^{M}{\left|g_{m}\left({\left(At\right)}_{m}-b_{m}\right)\right|}^{p}$$

where $G=\mathrm{diag}(g_{1},g_{2},\ldots ,g_{M})$, $g_{m}=(\gamma_{m}^{1/\alpha }∥t-r_{m}{∥)}^{-1}$, ${\left(At\right)}_{m}$ and $b_{m}$ are the *m*-th element of At and b, respectively. When the measurement noise is small, we have $\sin n_{m}\approx n_{m}$. Then, $|g_{m}\left({\left(At\right)}_{m}-b_{m}\right){|}^{p}$ can be expressed approximately as

(25) $$|g_{m}\left({\left(At\right)}_{m}-b_{m}\right){|}^{p}\approx \gamma_{m}^{-p/\alpha }∥t-r_{m}{∥}^{-2}|{\tilde{\theta }}_{m}-\theta_{m}{|}^{\left(p-2\right)}{\left|{\left(Ap\right)}_{m}-b_{m}\right|}^{2}$$

The above interpretation suggests an iterative reweighted pseudolinear least-squares with the weight matrix for the *i*-th iteration given by

(26) $$W^{\left(i\right)}=\mathrm{diag}\left(\gamma_{1}^{-\frac{p}{2\alpha }}∥{\hat{t}}^{\left(i-1\right)}-r_{1}{∥}^{-1}{\left|{\tilde{\theta }}_{1}-{\hat{\theta }}_{1}^{\left(i-1\right)}\right|}^{\frac{p-2}{2}},\ldots ,\gamma_{M}^{-\frac{p}{2\alpha }}{∥{\hat{t}}^{\left(i-1\right)}-r_{M}∥}^{-1}{\left|{\tilde{\theta }}_{M}-{\hat{\theta }}_{M}^{\left(i-1\right)}\right|}^{\frac{p-2}{2}}\right)$$

where ${\hat{t}}^{\left(i-1\right)}$ and ${\hat{\theta }}_{m}^{\left(i-1\right)}$ denote the estimated source location and the *m*th bearing obtained from the previous iteration i−1. Using the weight matrix, the weight error for the *k*-th iteration is given by

(27) $${\left(W^{\left(i\right)}\xi^{\left(i\right)}\right)}^{T}W^{\left(i\right)}\xi^{\left(i\right)}=\sum_{m=1}^{M}{\left|g_{m}\left({\left(At\right)}_{m}-b_{m}\right)\right|}^{p}$$

The homotopy method can be applied in the iterations by starting with a value for *p* equals to 2 (the weight matrix is unit matrix) and decreasing it each iteration until it reaches the designated value. Thus,

(28) $$p^{\left(i\right)}=\max\left(p,\kappa p^{\left(i-1\right)}\right)$$

where κ<1 is the homotopy parameter. The source location can be estimated by performing least-squares estimation:

(29) $${\hat{t}}^{\left(i\right)}={\left({\left(W^{\left(i\right)}A\right)}^{T}W^{\left(i\right)}A\right)}^{-1}{\left(W^{\left(i\right)}A\right)}^{T}W^{\left(i\right)}b$$

The IRPLS algorithm is stopped when $∥{\hat{t}}^{\left(i-1\right)}-{\hat{t}}^{\left(i\right)}∥\leq \epsilon$, where ϵ is a tolerance parameter.

Finally, the whole process of the IRPLS algorithm is summarized in Algorithm 1.
**Algorithm 1** The IRPLS algorithm.1:**Initialization:** Set ${\hat{t}}^{\left(0\right)}={\hat{t}}_{\mathrm{PLE}}$.2:**for**i=0; i++**do**3: Compute the weight matrix using (31).4: Determine the value of *p* for each iteration using (28).5: Perform weighted least squares estimation using (29).6: if $∥{\hat{t}}^{\left(i-1\right)}-{\hat{t}}^{\left(i\right)}∥\leq \epsilon$, we obtain the final solution, and stop the iteration. Otherwise i=i+1, go to step 3.7:**end for**

The Lp-norm criterion ensures that the cost function in (24) gives less weight to large deviations, and therefore reduces the bias formed by pseudolinear errors with large residuals. However, the IRPLS algorithm implicitly assumes that only b is subjected to errors. This is not the case, since the system matrix A is corrupted with measurement noises as well. The correlation between A and b causes the IRPLS estimator to be inconsistent. In this subsection, we analyze such bias attributed to the correlation between A and b.

Let ${\hat{t}}^{*}$ denote the final solution of the IRPLS algorithm. This solution satisfies

(30) $${\hat{t}}^{*}={\left(A^{T}W\left({\hat{t}}^{*}\right)A\right)}^{-1}A^{T}W\left({\hat{t}}^{*}\right)b$$

where

(31) $$W\left({\hat{t}}^{*}\right)=\mathrm{diag}\left(\gamma_{1}^{-\frac{p}{\alpha }}∥{\hat{t}}^{*}-r_{1}{∥}^{-2}{\left|{\tilde{\theta }}_{1}-{\hat{\theta }}_{1}^{*}\right|}^{\left(p-2\right)},\ldots ,\gamma_{M}^{-\frac{p}{\alpha }}{∥{\hat{t}}^{*}-r_{M}∥}^{-2}{\left|{\tilde{\theta }}_{M}-{\hat{\theta }}_{M}^{*}\right|}^{\left(p-2\right)}\right)$$

and ${\hat{\theta }}_{m}^{*}$ denotes the *m*th bearing obtained from (5) by using ${\hat{t}}^{*}$.

According to (22) and (30), we can get

(32) $$\Delta t={\hat{t}}^{*}-t=-{\left(A^{T}W\left({\hat{t}}^{*}\right)A\right)}^{-1}A^{T}W\left({\hat{t}}^{*}\right)\xi$$

The analytical bias of IRPLS is given by [14]

(33) $$E\left\{\Delta t\right\}=-\frac{1}{p-1}E{\left\{\frac{A^{T}W\left(t\right)A}{M}\right\}}^{-1}E\left\{\frac{A^{T}W\left(t\right)\xi }{M}\right\}$$

where the weighting matrix W(t) is computed from (31) using true source location parameter t.

## 4. Total Lp-Norm Optimization

In this section, we present a method of TLPO for BOSL. The IRPLS algorithm only considers the deviation of the system vector b. But in fact, the system matrix A also has a measurement residual. The IRPLS algorithm will inevitably cause a large bias, because the system matrix A and the system vector b are statistically correlated.

### 4.1. Method Description

In order to improve the performance of the IRPLS algorithm and reduce the bias caused by the correlation between A and b, we develop the TLPO method in this subsection. Unlike the IRPLS method, the TLPO method uses the correction matrix ΔA and the correction vector Δb to compensate the system matrix A and system vector b, respectively. The normalized equation can be written as

(34) G(A+ΔA)t=G(b+Δb)

where ΔA is the perturbation matrix of A and Δb is the perturbation vector of b. Let K=G[A,b] and $v={\left[t,-1\right]}^{T}$ be the augmented matrix and vector, and Equation (34) can be rewritten as

(35) (K+Ω)v=0

where Ω=G[ΔA,Δb] is the error matrix.

In order to reduce the bias of IRPLS, we use the TLPO approach to minimize the perturbation matrix ΔA and perturbation vector Δb simultaneously, and the TLPO problem for BOTL can be formulated as

(36) $$\min_{E,t}{∥\Omega ∥}_{p},\;s.\;t.\;\left(K+\Omega \right)v=0$$

for 1<p<2. Note that if p=1, (36) becomes the total least absolute residual method, and if p=2, it is the well-known TLS method. To solve the TLPO problem conveniently, it is necessary to develop an equivalent form of (36). The following proposition holds.

**Proposition** **1.** *Given the TLPO problem defined in (36), the estimation of t from the minimization of ${∥\Omega ∥}_{p}$ subject to (K+Ω)v=0 is equivalent to*

(37) $$\min_{v}{∥Kv∥}_{p},\;s.\;t.\;{∥v∥}_{q}-1=0$$

*where $v={\left[t,-1\right]}^{T}$, $\frac{1}{p}+\frac{1}{q}=1$ and 1<p<α.*

**Proof.**  From the constraint (K+Ω)v=0, we have ${∥Kv∥}_{p}={∥\Omega v∥}_{p}$. Let $\omega_{m}^{T}$ denote the *m*th row of Ω. ${∥\Omega v∥}_{p}^{p}$ becomes

(38) $$\begin{array}{cc} {∥\Omega v∥}_{p}^{p} & =\sum_{m=1}^{M}{\left|\omega_{m}^{T}v\right|}^{p}\\ \; & \leq \sum_{m=1}^{M}{∥\omega_{m}∥}_{p}^{p}{∥v∥}_{q}^{p} \end{array}$$

for *p* and *q* satisfy the equation $\frac{1}{p}+\frac{1}{q}=1$ and 1<p<α. To derive (38), we used the properties of hölder’s inequality [34]. If the Lq-norm of v satisfies ${∥v∥}_{q}=1$, then the inequality (38) becomes ${∥\Omega v∥}_{p}\leq {∥\Omega ∥}_{p}$. Therefore, we can conclude that the minimization of ${∥\Omega ∥}_{p}$ subject to (K+Ω)v=0 is equivalent to (37). □

Proposition 1 provides a facilitated way to solve the TLPO problem. In the following two subsections, we concentrate on deriving the solution on this particular problem and developing the IGED and GLM algorithms.

### 4.2. The IGED Algorithm

The optimization problem in Proposition 1 is equivalent to

(39) $$\min_{v}{∥Kv∥}_{p}^{p},\;s.\;t.\;{∥v∥}_{q}^{q}-1=0$$

To solve (39), we first transform it into an unconstrained optimization problem by using the Lagrange multiplier method. The appropriate Lagrangian function of (39) can be written as

(40) $$\begin{array}{cc} L(v,\lambda ) & ={∥Kv∥}_{p}^{p}{+\lambda (1-∥v∥}_{q}^{q})\\ \; & =v^{T}K^{T}DKv+\lambda \left(1-v^{T}Cv\right) \end{array}$$

where $D=\mathrm{diag}(|{\left(Kv\right)}_{m}{|}^{p-2})$, ${\left(Kv\right)}_{m}$ represents the *m*th element of vector Kv for m=1,2,…,M, $C=\mathrm{diag}(|v_{1}^{q-2}|,|v_{2}^{q-2}|,|v_{3}^{q-2}|)$ and λ is the Lagrangian multiplier. Taking the partial derivative of L(v,λ) with respect to v and setting it to zero yields

(41) $$K^{T}DKv=\lambda Cv$$

After multiplying both sides of (41) with $v^{T}$, it can be found that only $\lambda =v^{T}K^{T}DKv$ is the cost to be minimized. So, the optimal solution $v^{*}$ is the eigenvector of the smallest generalized eigenvalue of $\left(K^{T}DK,C\right)$.

**Figure 2:**
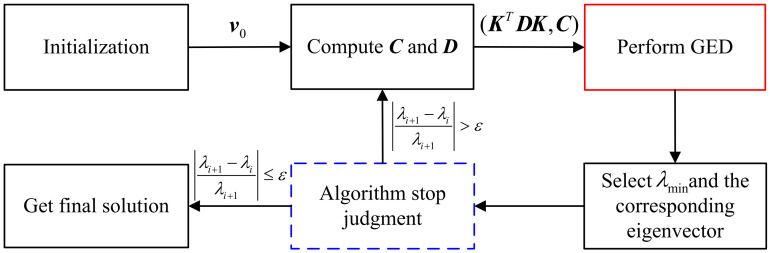
The flowchart of the IGED algorithm.

Figure 2 gives a flowchart to depict the whole process of the IGED algorithm. The algorithm starts from the initialization procedure, where $v_{0}$ is set as ${\left[{\hat{t}}_{\mathrm{PLE}}^{T},-1\right]}^{T}$ and ${\hat{t}}_{\mathrm{PLE}}$ is obtained from (23). Given *p* and *q*, matrices C and D can be computed, respectively. After performing generalized eigenvalue decomposition from the pair $\left(K^{T}DK,C\right)$, the solution of v in (41) is the generalized eigenvector of $\left(K^{T}DK,C\right)$ that gives the minimum generalized eigenvalue. Since IGED is an iterative algorithm, it requires the convergence check. Let $\lambda_{i}$ and $\lambda_{i+1}$ be the minimum generalized eigenvalues at the *i*th and i+1th iterations, respectively. If there exists ε such that

(42) $$\left|\frac{\lambda_{i+1}-\lambda_{i}}{\lambda_{i+1}}\right|\leq \varepsilon$$

then we would get the final solution $v^{*}$ and the estimate for source location is

(43) $$\hat{t}=\frac{v^{*}\left(1:2\right)}{v^{*}\left(3\right)}.$$

In summary, the IGED Algorithm 2 can be implemented by the following procedures.
**Algorithm 2** The IGED algorithm.1:**Initialization:**$v_{0}={\left[{\hat{t}}_{\mathrm{PLE}}^{T},-1\right]}^{T}$.2:**for**i=0; i++**do**3: Compute C and D.4: Perform generalized eigenvalue decomposition of the pair $\left(K^{T}DK,C\right)$.5: Select the smallest generalized eigenvalue and its corresponding generalized eigenvector.6: Set $\lambda_{i}$ and $\lambda_{i+1}$ as the minimum generalized eigenvalues for iterations *i* and i+1. If $|\left(\lambda_{i+1}-\lambda_{i}\right)/\lambda_{i+1}|>\varepsilon$, go to step 3. Otherwise, obtain the final solution $v^{*}$ and stop.7: Get the source location estimate $\hat{t}$ using (43).8:**end for**

### 4.3. The GLM Algorithm

The GLM method [35] is widely used for constrained optimization. Its basic principle is to add a penalty term to the Lagrangian function to form an augmented Lagrangian function, which can impose a larger penalty on infeasible points. Thus, the constrained optimization problem (37) would be transformed into a new unconstrained optimization problem that can be solved efficiently.

To solve (37) with the GLM method, we formulate an augmented Lagrangian function as follows:

(44) $$L\left(v,\lambda ,s\right)=g\left(v\right)+\frac{s}{2}{∥h\left(v\right)∥}_{2}^{2}-\lambda h(v)$$

where $g\left(v\right)={∥Kv∥}_{p}$ and $h\left(v\right)=1-{∥v∥}_{q}$. Compared with the Lagrangian function, the GLM cost function has an additional term $s/{2∥h\left(v\right)∥}_{2}^{2}$. This item is a punishment for violation of constraint h(v)=0. The punishment parameter *s* determines the degree of punishment, and it is generally sufficiently large. λ is a positive scalar, and the term λh(v) is to ensure that the optimal solution is a strict local minimum point of $F\left(v\right)=g\left(v\right)+\frac{s}{2}{∥h\left(v\right)∥}_{2}^{2}$ under the condition of obeying the constraint h(v)=0.

**Figure 3:**
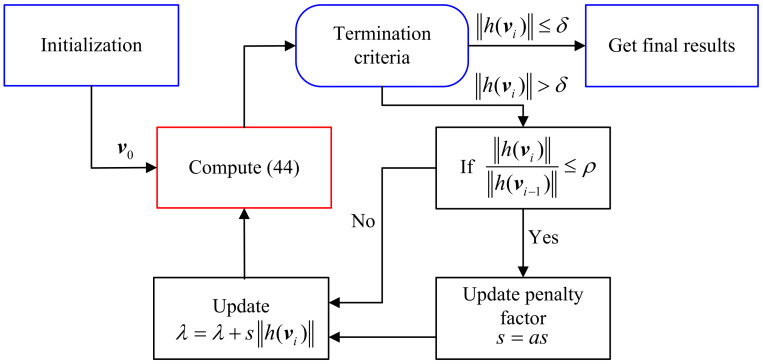
The flow diagram of the GLM algorithm.

Figure 3 shows the flow diagram of the GLM algorithm. For the initialization step, we set $v_{0}={\left[{\hat{t}}_{\mathrm{PLE}}^{T},-1\right]}^{T}$. Given initial multiplier λ, penalty factor *s*, admissible error ε, scaling parameter a>1 and parameter ρ∈(0,1), the problem (44) can be solved by using the existing unconstrained optimization methods, such as Newton, Interior point method, simplex method, etc. We use fminsearch function in MATLAB to estimate v from (44). Then, we check whether the termination criteria are satisfied. If $∥h(v_{i})∥\leq \delta$, the iteration terminates and $v_{i}$ is the near optimal solution of (37). Otherwise, go to the next step which is to control the convergence speed. If there exists ρ∈(0,1) such that

(45) $$\frac{∥h(v_{i})∥}{∥h(v_{i-1})∥}\geq \rho$$

we can increase the penalty factor *s* by setting s=a·s (a>1). If not, then we leave the value of *s* unchanged. The penalty factor is used for multiplier update $\lambda =\lambda +s∥h(v_{i})∥$. The updated λ and *s* are used to solve the unconstrained optimization problem (44) again.

Finally, the implementation process of the GLM algorithm is summarized in Algorithm 3.
**Algorithm 3** The GLM algorithm.1:**Initialization:**$v_{0}={\left[{\hat{t}}_{\mathrm{PLE}}^{T},-1\right]}^{T}$, δ, λ, *s*, a>1, ρ∈(0,1).2:**for**i=0; i++**do**3: Using $v_{i}$ as the initial point, find the minimum value of L(v,λ,s).4: If $∥h(v_{i})∥\leq \delta$ holds, stop the iterations and get the final solution $v^{*}$, and go to step 7. Otherwise, go to step 5.5: If $∥h\left(v_{i}\right)∥/∥h\left(v_{i-1}\right)∥\geq \rho$, update the penalty coefficient s=a·s, and go to step 6. Otherwise, go to step 6 directly.6: Update $\lambda =\lambda -\rho ∥h(v_{i})∥$ and go to step 3.7: Find the target position $\hat{t}=v^{*}\left(1:2\right)/v^{*}\left(3\right)$.8:**end for**

### 4.4. Computational Complexity Analysis

In this subsection, we analyze the computational complexities of the proposed algorithms and compare with those of PLE and IRPLS. We only consider the asymptotic computational cost. For each algorithm, we separate the complexity calculation to small steps, e.g., multiplying two matrices of size n×m and m×p costs $O\left(nmp\right)$. Let $L_{1}$, $L_{2}$ and $L_{3}$ denote the number of iterations with regarding to IRPLS, IGED and GLM, respectively. The iterative implementation of IRPLS, IGED and GLM, however, could be very sensitive to initialization. In this paper, we use the solution of PLE for initialization.

The computational costs are listed in Table 1. Table 1 summarizes the computational complexities of the PLE and IRPLS, IGED and GLM algorithms. PLE requires the least amounts of computation. IRPLS and IGED exhibit the similar computational costs. For the GLM algorithm, we employ the Broyden–Fletcher–Goldfarb–Shanno (BFGS) [33] algorithm to solve the unconstrained problem presented in (44). BFGS is an iterative method for solving unconstrained nonlinear optimization problems by providing an approximation to the Hessian matrix. We assume that the number of iterations for BFGS is $N_{i}$ for the *i*th iteration of GLM. For each iteration, we assume the order of computational complexity of BFGS is $O_{i}\left(M\right)$. The GLM algorithm needs to estimate v which is computationally demanding. The estimation procedure needs to repeat $L_{3}$ times since it is iterative.

## 5. Performance Bound

In this section, we present the Cramér-Rao Lower Bound (CRLB) for robust BOSL when the measurement noise is modeled as SαS distribution. We then discuss how the CRLB is calculated. The CRLB for any unbiased estimator of t is derived by the inverse of the Fisher Information Matrix (FIM) [36], which is computed as

(46) $$\mathrm{CRLB}\left(t\right)=J^{-1}$$

where J is the FIM given by the 2×2 matrix

(47) $$J=E\left\{\left[\begin{array}{cc} {\left(\frac{\partial f(\tilde{\phi }|t)}{\partial t_{x}}\right)}^{2} & \frac{\partial f(\tilde{\phi }|t)}{\partial t_{x}}\frac{\partial f(\tilde{\phi }|t)}{\partial t_{y}}\\ \frac{\partial f(\tilde{\phi }|t)}{\partial t_{x}}\frac{\partial f(\tilde{\phi }|t)}{\partial t_{y}} & {\left(\frac{\partial f(\tilde{\phi }|t)}{\partial t_{y}}\right)}^{2} \end{array}\right]\right\}$$

where $f(\tilde{\phi }|t)$ denotes the Probability Density Function (PDF) of $\tilde{\phi }$. Since $e_{1},e_{2},\ldots ,e_{M}$ have the same standard dispersion, the PDF of ${\tilde{\phi }}_{m}$ is

(48) $$f_{\alpha }\left(e\right)=f_{\alpha }\left({\tilde{\phi }}_{m}-\phi_{m}\right)=\frac{1}{2\pi }\int_{-\infty }^{+\infty }\exp\left(-i\left({\tilde{\phi }}_{m}-\phi_{m}\right)\tau -\gamma {\left|\tau \right|}^{\alpha }\right)d\tau$$

According to [37], the FIM J for the parameters $t_{x}$ and $t_{y}$ is given by

(49) $$J=\kappa_{\alpha }\sum_{m=1}^{M}\left[\begin{array}{cc} {\left(\frac{\partial \phi_{m}}{\partial t_{x}}\right)}^{2} & \frac{\partial \phi_{m}}{\partial t_{x}}\frac{\partial \phi_{m}}{\partial t_{y}}\\ \frac{\partial \phi_{m}}{\partial t_{x}}\frac{\partial \phi_{m}}{\partial t_{y}} & {\left(\frac{\partial \phi_{m}}{\partial t_{y}}\right)}^{2} \end{array}\right]$$

where $\kappa_{\alpha }$ is the Fisher information for the location of $f_{\alpha }\left(e\right)$,

(50) $$\kappa_{\alpha }=\int_{-\infty }^{+\infty }\frac{{\left(f_{\alpha }^{\prime }\left(e\right)\right)}^{2}}{f_{\alpha }\left(e\right)}de$$

and the expressions of $\frac{\partial \phi_{m}}{\partial t_{x}}$ and $\frac{\partial \phi_{m}}{\partial t_{x}}$ are given by

(51) $$\frac{\partial \phi_{m}}{\partial t_{x}}=-\frac{t_{y}-r_{y,m}}{∥t-r_{m}∥},\;\frac{\partial \phi_{m}}{\partial t_{y}}=\frac{t_{x}-r_{x,m}}{∥t-r_{m}∥}$$

Note that the value of $\kappa_{\alpha }$ depends on the parameter α. If α=1, $f_{\alpha }\left(e\right)$ follows a Cauchy PDF and $\kappa_{\alpha }$ equals to $\frac{3}{5}$ for γ=1. In particular, when α=2, $f_{\alpha }\left(e\right)$ is Gaussian with variance 2γ and $\kappa_{\alpha }=\gamma$. Therefore, the CRLB of t in the case of α=2 is consistent with the CRLB result given in [8].

However, when 1<α<2, $f_{\alpha }\left(e\right)$ has no closed-form expressions. Hence, computing $f_{\alpha }\left(e\right)$ involves numerically evaluating the integrals in (48). We use the MATLAB function *STBLPDF* to compute the pdf of the SαS distribution. The derivative of $f_{\alpha }\left(e\right)$ is also calculated by using numerical differentiation methods.

## 6. Simulations

In this section, we present three simulation examples to illustrate the performance of the proposed IGED and GLM algorithms and compare them with the PLE and IRPLS algorithm, as well as the CRLB. The localization performance is characterized with the bias norm and the root-mean-square-error (RMSE). The RMSE for source localization is defined as

(52) $$\mathrm{RMSE}={\left(\frac{1}{N}\sum_{n=1}^{N}{∥{\hat{t}}_{n}-t∥}^{2}\right)}^{1/2}$$

where *N* represents the total number of Monte Carlo runs, and ${\hat{t}}_{n}$ denotes the source location estimate at the *n*th Monte Carlo run. The expression of the bias norm is given by

(53) $$\mathrm{BIAS}=∥\frac{1}{N}\sum_{n=1}^{N}\left({\hat{t}}_{n}-t\right)∥$$

The RMSE given in (52) is bounded by the square root of the trace of the Cramér-Rao Lower Bound matrix

(54) $$\mathrm{RMSE}\geq \sqrt{tr(\mathrm{CRLB}(t))}$$

where tr(·) denotes the trace of a matrix.

In the simulations, we examine the localization accuracy versus three kinds of parameters: (1) noise dispersion; (2) number of sensors; and (3) noise impulsiveness. All the sensors are uniformly placed in a 100×100 $m^{2}$ plane centered at (50,50) m. The source is located at (100,100) m. The IRPLS algorithm and the proposed IGED and GLM algorithms are iterative and they are all initialized by the PLE derived from (23) for ensuring a fair comparison. The termination parameters ϵ, ε and δ are set as $\epsilon =10^{-5}$, $\varepsilon =10^{-10}$ and $\delta =10^{-5}$, respectively. For IRPLS, IGED and GLM, the maximum iteration is fixed at 200.

### 6.1. Various Levels of Noise Dispersion

In this example, we illustrate the RMSE and bias performance of PLE, IRPLS, IGED and GLM versus noise dispersion. For convenience, we use the generalized signal-to-noise ratio (GSNR) to characterize different noise levels. The GSNR is inversely proportional to the noise dispersion γ and is calculated as 1/γ after normalized. The range of $\gamma^{1/\alpha }$ is from 2π/180 radian to 6π/180 radian. The characteristic exponent α is set to 1.5 and the corresponding optimum value of *p* is 1.225.

**Figure 4:**
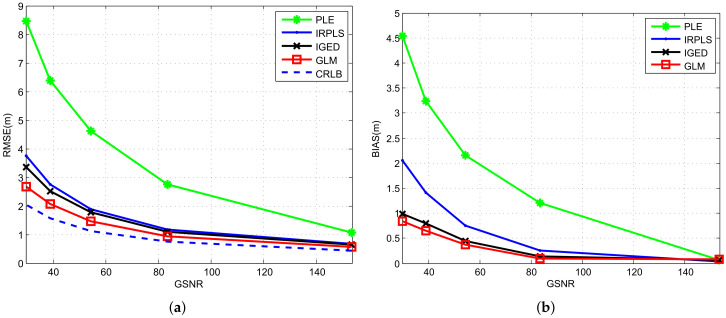
RMSE and bias performance comparison of PLE, IRPLS, IGED and GLM estimates with various GSNRs. (**a**) RMSE results. (**b**) Bias norm results.

Figure 4 plots the RMSE and bias curves of the PLE, IRPLS, IGED and GLM algorithms together with the CRLB. For RMSE curves shown in Figure 4a, the green line with Asterisk is the RMSE value for the PLE algorithm, the blue line with Point is for IRPLS, the black line with Cross is for IGED, the red line with Square is for GLM and the blue dash line is the CRLB. The PLE method can not give accurate target location estimates in the presence of impulsive noise. The large bias norm formed by the outlier data in turn leads to a poor performance for the PLE. The least-norm estimators, including IRPLS, IGED and GLM, yield much better localization performance. However, the IRPLS method still has a large estimation bias because it cannot overcome the bias attributed to the correlation between the system matrix A and the data vector b. On the other hand, the IGED and GLM algorithms utilizes the total Lp-norm optimization technique that can minimize the errors in A and b simultaneously. Therefore, the IGED and GLM algorithms are capable of outperforming the IRPLS in much lower RMSEs and biases.

**Figure 5:**
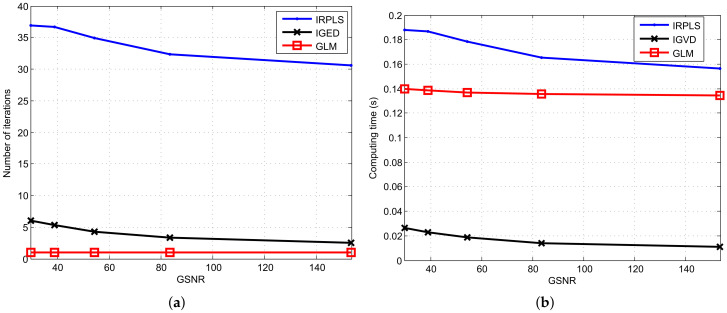
Number of iterations and computing time versus GSNR for the IRPLS, IGED and GLM algorithms. (**a**) Number of iterations. (**b**) Computing time.

Beyond the RMSE and bias performance, we need to explore the number of iterations and the computation time of IRPLS, IGED and GLM. The results are depicted in Figure 5. The algorithms run on a laptop with CPU i5-7200U @ 2.5 GHz and RAM 8 GB. The version of software is MATLAB 2017a. Collectively, the average number of iterations decreases when the GSNR increases. The GLM appears to have the least number of iterations. Its average number of iterations reduces from 1.05 to 1.01 when the GSNR ranges from 29.51 to 153.3. However, its computation time for single iteration is high (i.e., 0.1343 s), because the GLM algorithm has the steps of unconstrained optimization. On the contrary, the IGED algorithm has the least computing time since its computational complexity mainly lies in generalized eigenvalue decomposition. The IRPLS algorithm has high computing time due to the large number of iterations (over 30 iterations).

### 6.2. Different Number of Sensors

**Figure 6:**
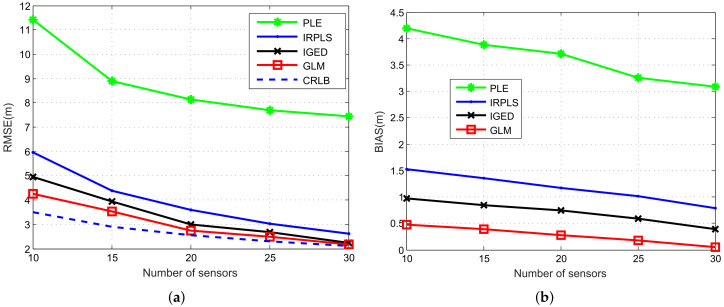
RMSE and bias performance comparison of PLE, IRPLS, IGED and GLM estimates with different number of sensors. (**a**) RMSE results. (**b**) Bias norm results.

**Figure 7:**
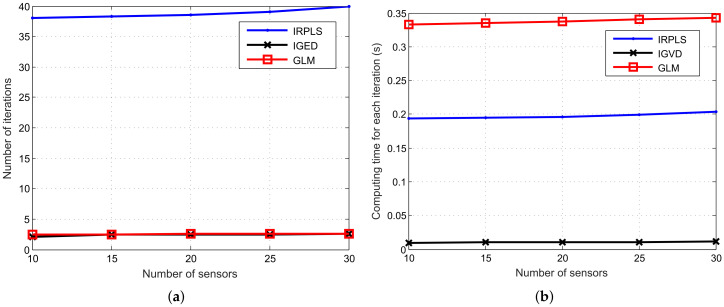
Number of iterations and computing time versus number of sensors for the IRPLS, IGED and GLM algorithms. (**a**) Number of iterations. (**b**) Computing time.

In this subsection, we compare the RMSE and bias performance of the PLE, IRPLS, IGED and GLM algorithms over the number of sensors *M* ranging from 10 to 30, five at a time, when $\gamma^{1/\alpha }$ is kept at 4π/180 radian. The other parameters remain the same. The simulation results are shown in Figure 6 and Figure 7.

From Figure 6, we observe that the GLM algorithm has better RMSE and bias performance than that of PLE, IRPLS and IGED. When M≥20, the RMSE of GLM is close to the CRLB. The IGED algorithm slightly deviates from the CRLB, because the algorithm has not fully converged. Moreover, we also observe from Figure 7 that the average iterations is kept at 2.5 and is almost the same as that of the GLM algorithm. Meanwhile, the bias of IGED is higher than that of GLM. Setting more iterations would help the IGED algorithm achieve better performance. As expected, the PLE exhibits unreliable results since it is not robust to the impulsive noise. The bias of IRPLE does not vanish as the number of sensors increases. This phenomenon is also validated in (33). In terms of computational complexity, the total computing time for GLM is about 0.3378 s when the number of sensors is fixed at 20. The IGED has the least amounts of computation and it only requires 0.01032 s at N=20.

### 6.3. Various Values of Noise Impulsiveness

To further verify the effectiveness of the proposed algorithms, we examine the performance of the algorithms for different levels of noise impulsiveness. In the following figures drawing the simulation results, the value of the noise impulsiveness level α deviates from 1.9 to 1.1 and the corresponding optimum values of *p* are set as 1.546, 1.430, 1.348, 1.282, 1.225, 1.174, 1.127, 1.083, 1.041 from (17). In this example, $\gamma^{1/\alpha }$ is fixed at 4π/180 radian. The other parameters remain unchanged.

**Figure 8:**
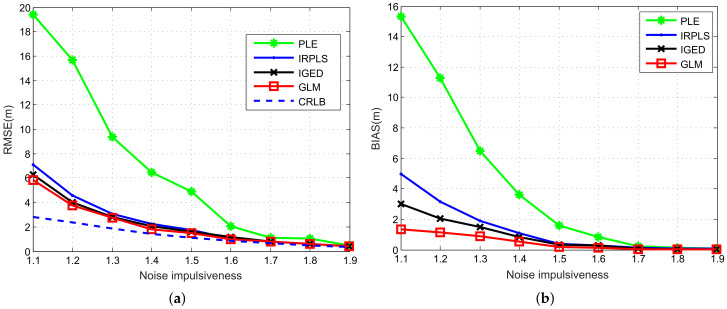
RMSE and bias performance comparison of PLE, IRPLS, IGED and GLM estimates with various noise impulsiveness. (**a**) RMSE results. (**b**) Bias norm results.

**Figure 9:**
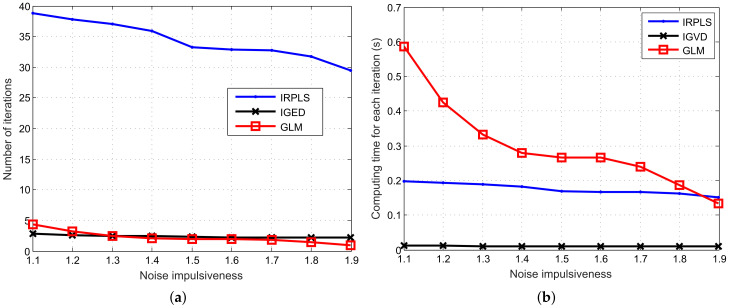
Number of iterations and computing time versus noise impulsiveness for the IRPLS, IGED and GLM algorithms. (**a**) Number of iterations. (**b**) Computing time.

Figure 8 depicts the RMSE and bias performance with respect to various α. As shown in Figure 8, the RMSE and the bias of PLE is much higher than that of IRPLS, IGED and GLM, which is caused by the impulsive noise. As α decreases, the influence of the noise impulsiveness becomes more significant. The level of noise impulsiveness also affects the bias and RMSE performance of IRPLS. The GLM algorithm has relatively small bias and its RMSE is very close to the CRLB when α≥1.4. The IGED has the comparable RMSE performance with the GLM. However, the bias of IGED slightly deviates from that of GLM as α decreases. From Figure 9, we observe that the number of iterations of IRPLS, GLM and IGED decrease as α increases, i.e., the number of average iterations reduces from 38.8 to 29.5 for IRPLS, from 4.4 to 1 for GLM and from 2.9 to 2.2 for IGED. The computation time also decreases when α increases. Again, the computation time of GLM is much higher than that of IGED.

### 6.4. Different Number of Iterations

**Figure 10:**
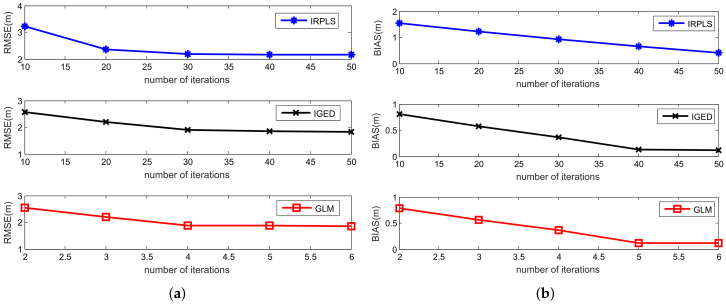
RMSE and bias performance versus the number of iterations for IRPLS, IGED and GLM. (**a**) RMSE results. (**b**) Bias norm results.

In order to further verify the influence of the number of iterations on the proposed algorithm, we tested the performance of the algorithm for the increasing number of iterations. The simulation results are shown in Figure 10, where the number of iterations is set 50 for IRPLS and IGED, and 6 for GLM. The RMSE and bias are recorded every 10 iterations for IRPLS and IGED. The RMSE and bias performance of GLM is plotted for each iteration. In this example, $\gamma^{1/\alpha }$ is fixed at 4π/180 radian. The number of sensors is kept at 20. The characteristic exponent α is set to 1.5 and the corresponding optimum value of *p* is 1.225.

Figure 10 describes the RMSE and bias performance of the IRPLS, IGED and GLM algorithms versus different iterations. As shown in Figure 10a, the RMSE result of IRPLS remains at 2.1 m after 30 iterations. We observe that the bias of the IRPLS algorithm decreases as the number of iterations increasing, as shown in Figure 10b. However, the bias does not vanish. It still has 0.41 m after 50 iterations. The RMSE value of IGED keeps at 1.8 m after 30 iterations and the bias of IGED reduces to 0.12 m using about 40 iterations. Differing from the IRPLS and IGED algorithms, the RMSE result of GLM reaches at 1.8 m using only four iterations. After 5 iteratios, the bias of GLM reduces to 0.12 m. From Figure 10, we can observe that the RMSE and bias performance of IGED is almost the same as that of GLM if IGED runs a sufficient number of iterations.

### 6.5. Scalability Evaluation

We analyze the scalability of the IRPLS, IGED and GLM algorithms in this subsection for increasing number of sensors, from 20 to 100, and decreasing levels of noise impulsiveness, from α=1.1 to α=1.9. To examine the scalability of the iterative algorithms, the IRPLS, IGED and GLM algorithms share the same stopping condition. Let ${\hat{t}}_{i}$ denote estimation result for the *i*th iteration. The criterion for stopping these iterative algorithms is given by $∥{\hat{t}}_{i}-{\hat{t}}_{i-1}∥<10^{-5}$.

**Figure 11:**
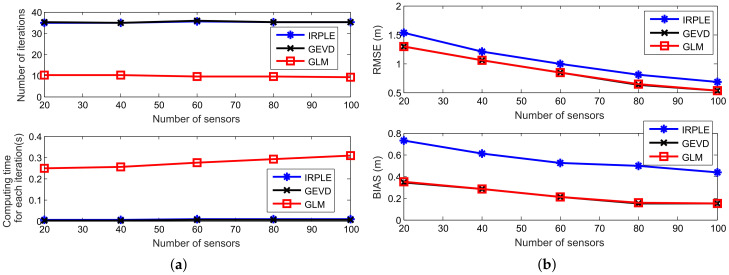
Scalability analysis for various number of sensors. (**a**) Number of iterations and computing time. (**b**) RMSE and bias performance.

Figure 11 compares the number of iterations, computing time for each iteration, RMSE and bias performance of the IRPLS, IGED and GLM algorithms versus different numbers of sensors. The other parameters remain the same as Section 6.4. It can be observed from Figure 11 that the number of iterations of these algorithms does not reduce as the number of sensors increases. Among them, the number of iterations for IRPLS and IGED maintains at 35, while that of the GLM algorithm keeps at 10. The computing time for each iteration, however, slightly increases as the number of sensors increasing. The RMSE and bias performance decreases as the number of sensors increases due to the fact that more normal sensors can be utilized. This result is also demonstrated in Figure 6. We also observe that the RMSE and bias curves of IGED and GLM are consistently lower than those of IRPLS, which shows the performance advantage of the proposed algorithms.

**Figure 12:**
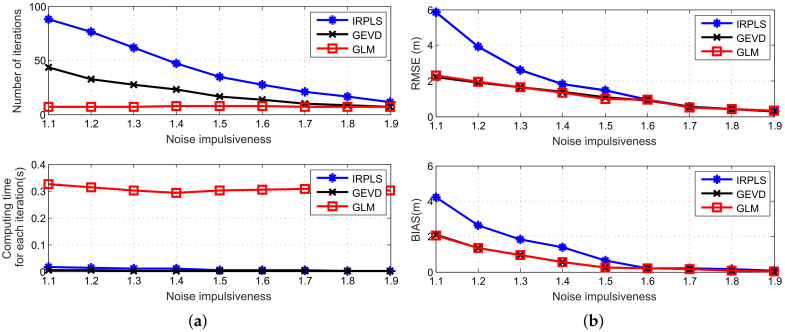
Scalability analysis for different levels of noise impulsiveness. (**a**) Number of iterations and computing time. (**b**) RMSE and bias performance.

Figure 12 shows the number of iterations, computing time for each iteration, RMSE and bias performance of the IRPLS, IGED and GLM algorithms versus different levels of noise impulsiveness. The number of sensors is set at 20. Other parameters remain unchanged. From Figure 12a, we observe that the number of iterations for IRPLS scales from 88 to 11, 43 to 7 for IGED. However, the iteration value does not change for GLM and the number keeps at 7. That is because GLM performs optimization at each iteration to reduce the impact of impulsive noise. Moreover, the RMSE and bias performance of these algorithms decreases as increasing the value of α. As expected, IGED and GLM exhibit almost the same performance. A small value of α results in a poorer IRPLE performance, as illustrated in Figure 12b. When α is greater than 1.6, the RMSE and bias curves of IRPLS reach those of IGED and GLM due to the fact that the level of noise impulsiveness has less impact on the bias performance.

## 7. Conclusions

In this paper, a total Lp-norm optimization method is presented to solve the BOSL problem and two algorithms, named IGED and GLM, are proposed to fulfill the optimization of the total least Lp-norm. By minimizing the errors in the system matrix and the data vector simultaneously for the least Lp-norm optimization, the proposed algorithms can overcome the bias arising from the correlation between the system matrix and the pseudolinear noise vector.

Simulation results show that the proposed IGED and GLM algorithms have much better RMSE and bias performance than the IRPLS algorithm. The number of iterations for GLM remains at a low level. With only a few iterations, the RMSEs of GLM can approach the CRLB. However, GLM expends more computation time than IGED under the same number of iterations, since GLM requires unconstrained optimization in each iteration.

## Figures and Tables

**Table 1:** Computational complexity.

Method	Operation	Cost
PLE	$A^{T}A$, $A^{T}b$	O(4M)+O(2M)
	${\left(A^{T}A\right)}^{-1}$	$O(2^{3})$
	${\left(A^{T}A\right)}^{-1}A^{T}b$	O(4M)
IRPLS	Compute $W^{\left(i\right)}A$ $L_{1}$ times	$O(2L_{1}M)$
	Compute $W^{\left(i\right)}b$ $L_{1}$ times	$O(L_{1}M)$
	Compute (29) $L_{1}$ times	$O(L_{1}\left(10M+8\right))$
IGED	Compute DK $L_{2}$ times	$O(3L_{2}M)$
	Perform GED $L_{2}$ times	$O(3^{3}L_{2})$
GLM	$L_{3}$ evaluations of BFGS	$O(\sum_{i=1}^{L_{3}}N_{i}O_{i}\left(M\right))$

## Data Availability

Not applicable.

## References

[1] Li X., Luo X., Zhao S. (2020). Globally Convergent Distributed Network Localization Using Locally Measured Bearings. IEEE Trans. Control. Netw. Syst..

[2] Kaplan L.M., Le Q. (2005). On exploiting propagation delays for passive target localization using bearings-only measurements. J. Frankl. Inst..

[3] Volgyesi P., Balogh G., Nadas A., Nash C.B., Ledeczi A. Shooter localization and weapon classification with soldier-wearable networked sensors. Proceedings of the International Conference on Mobile Systems, Applications and Services.

[4] Ali A.M., Yao K., Collier T.C., Taylor C.E., Blumstein D.T., Girod L. (2009). An empirical study of collaborative acoustic source localization. J. Signal Process. Syst..

[5] Wang Y., Wang G., Chen S., Ho K.C., Huang L. (2020). An Investigation and Solution of Angle Based Rigid Body Localization. IEEE Trans. Signal Process..

[6] Doğançay K. (2006). Bias compensation for the bearings-only pseudolinear target track estimator. IEEE Trans. Signal Process..

[7] Wang Y., Ho K.C. (2015). An Asymptotically Efficient Estimator in Closed-Form for 3-D AOA Localization Using a Sensor Network. IEEE Trans. Wirel. Commun..

[8] Gavish M., Weiss A.J. (1992). Performance analysis of bearing-only target location algorithms. IEEE Trans. Aerosp. Electron. Syst..

[9] Luo J.A., Shao X.H., Peng D.L., Zhang X.P. (2019). A Novel Subspace Approach for Bearing-Only Target Localization. IEEE Sens. J..

[10] Swami A., Sadler B.M. TDE, DOA and related parameter estimation problems in impulsive noise. Proceedings of the IEEE Signal Processing Workshop on Higher-Order Statistics.

[11] Kozick R.J., Sadler B.M. (2000). Maximum-likelihood array processing in non-Gaussian noise with Gaussian mixtures. IEEE Trans. Signal Process..

[12] Oh H., Seo D., Nam H. (2020). Design of a Test for Detecting the Presence of Impulsive Noise. Sensors.

[13] Shao M., Nikias C.L. (1993). Signal processing with fractional lower order moments: Stable processes and their applications. Proc. IEEE.

[14] Nguyen N.H., Doğançay K., Kuruoğlu E.E. (2019). An Iteratively Reweighted Instrumental-Variable Estimator for Robust 3-D AOA Localization in Impulsive Noise. IEEE Trans. Signal Process..

[15] Zhong X., Premkumar A.B., Madhukumar A.S. (2013). Particle Filtering for Acoustic Source Tracking in Impulsive Noise with Alpha-Stable Process. IEEE Sens. J..

[16] Tsakalides P., Nikias C.L. (1995). Maximum likelihood localization of sources in noise modeled as a stable process. IEEE Trans. Signal Process..

[17] Luo J.A., Fang F., Shi Y.F., Shen-Tu H., Guo Y.F. L1-Norm and Lp-Norm Optimization for Bearing-Only Positioning in Presence of Unreliable Measurements. Proceedings of the Chinese Control And Decision Conference (CCDC).

[18] Maronna B.R.A., Martin D.R., Yohai V.J. (2006). Robust Statistics: Theory and Methods.

[19] Liu Y., Hu Y.H., Pan Q. (2012). Distributed, robust acoustic source localization in a wireless sensor network. IEEE Trans. Signal Process..

[20] Jiang Y., Azimi-Sadjadi M.R. A robust source localization algorithm applied to acoustic sensor network. Proceedings of the IEEE International Conference on Acoustics, Speech and Signal Processing.

[21] Panigrahi T., Panda G., Mulgrew B., Majhi B. Robust incremental LMS over wireless sensor network in impulsive noise. Proceedings of the International Conference on Computational Intelligence and Communication Networks.

[22] Luo J.A., Xue C.C., Peng D.L. (2020). Robust Bearing-Only Localization Using Total Least Absolute Residuals Optimization. Complexity.

[23] Satar B., Soysal G., Jiang X., Efe M., Kirubarajan T. (2020). Robust Weighted *l*_1,2_ Norm Filtering in Passive Radar Systems. Sensors.

[24] Chen Y., So H.C., Kuruoglu E.E. (2015). Variance analysis of unbiased least lp-norm estimator in non-Gaussian noise. Signal Process..

[25] Jiang X., Chen J., So H.C., Liu X. (2018). Large-Scale Robust Beamforming via *ℓ*_∞_-Minimization. IEEE Trans. Signal Process..

[26] Wu H., Chen S.X., Zhang Y.H., Zhang H.Y., Ni J. (2015). Robust structured total least squares algorithm for passive location. J. Syst. Eng. Electron..

[27] Picard J.S., Weiss A.J. (2010). Bounds on the number of identifiable outliers in source localization by linear programming. IEEE Trans. Signal Process..

[28] Fragkos G., Apostolopoulos P.A., Tsiropoulou E.E. (2019). ESCAPE: Evacuation strategy through clustering and autonomous operation in public safety systems. Future Internet.

[29] Zhang Y., Guizani M. (2011). Game Theory for Wireless Communications and Networking.

[30] Cambanis S., Miller G. (1981). Linear Problems in Linear Problems in pth Order and Stable Processes. SIAM J. Appl. Math..

[31] Mallick M. (2018). A Note on Bearing Measurement Model; ResearchGate. https://www.researchgate.net/publication/325214760_A_Note_on_Bearing_Measurement_Model.

[32] Bishop A.N., Anderson B.D.O., Fidan B., Pathirana P.N., Mao G. (2009). Bearing-Only Localization using Geometrically Constrained Optimization. IEEE Trans. Aerosp. Electron. Syst..

[33] Roger F. (1987). Practical Methods of Optimization.

[34] Grinshpan A.Z. (2010). Weighted inequalities and negative binomials. Adv. Appl. Math..

[35] Hestenes M.R. (1969). Multiplier and gradient methods. Optim. Theory Appl..

[36] Kay S.M. (1993). Fundamentals of Statistical Signal Processing: Estimation Theory.

[37] Sadler B.M., Kozick R.J., Moore T. Performance analysis for direction finding in non-Gaussian noise. Proceedings of the IEEE International Conference on Acoustics, Speech, and Signal Processing.

